# Events and children’s sense of time: a perspective on the origins of everyday time-keeping

**DOI:** 10.3389/fpsyg.2015.00259

**Published:** 2015-03-11

**Authors:** Helen Forman

**Affiliations:** ^1^Department of Psychology, University of UmeåUmeå, Sweden

**Keywords:** sense of time, time-keeping, temporal organization, event knowledge, events, time perception, event perception, children

## Abstract

In this article I discuss abstract or pure time versus the content of time, (i.e., events, activities, and other goings-on). Or, more specifically, the utility of these two sorts of time in time-keeping or temporal organization. It is often assumed that abstract, uniform, and objective time is a universal physical entity *out there*, which humans may perceive of. However, this sort of evenly flowing time was only recently introduced to the human community, together with the mechanical clock. Before the introduction of mechanical clock-time, there were only events available to denote the extent of time. Events defined time, unlike the way time may define events in our present day culture. It is therefore conceivable that our primeval or natural mode of time-keeping involves the perception, estimation, and coordination of events. I find it likely that events continues to subserve our sense of time and time-keeping efforts, especially for children who have not yet mastered the use of clock-time. Instead of seeing events as a distraction to our perception of time, I suggest that our experience and understanding of time emerges from our perception of events.

## Introduction

The ability to keep track of events, activities, and other *goings-on* in our environment is of fundamental importance for our adaptation to the conditions of our earthly habitat. In everyday life, we need to organize and coordinate our own activities with that of others in our community. This ability for perceiving the constellation of events around us, how they are configured in relation to each other as well as to ourselves, is what makes the cross-temporal organization of our everyday lives at all possible. Both the ability to perceive these events and the ability to organize our own activities in concordance with the configuration of these events, is often referred to as having a *Sense of time.* Time in itself is not the main objective, though, but the events that may be gauged in terms of their temporal extent. This is so, because every event has a temporal aspect. *Cross-temporal organization of behavior* would perhaps, be another way of describing this ability, but I have chosen the shorter term *time-keeping*.

Initially my project was to investigate the ability for time-keeping in children, but for reasons explicated in the following account, I found it necessary to take a closer look at time-keeping generally.

In our present day culture we have a magnificent tool for time keeping at our disposal in the form of standardized time units or clock-time. The duration of any event or activity can be translated into uniform and objective time units. This way, events may be measured, added up and compared, forward, backward, and sideways, any way you like, in a perfectly objective and reliable manner. The process is somewhat analogous to how we use money for reasoning about and carrying out transactions concerning value. Money is a token of value or an abstraction of value. Any and every traded commodity may be translated into the abstract value of money. In the same way we may reason about and carry out transactions involving events and activities in terms of standardized clock-time.

Children, however, do not have access to this tool as their skills in time-keeping by means of clock-time is limited. Even though they learn how to read a clock, to tell time, during their early school years, it takes them a long time to learn to translate their experience into standardized time units (Harner, 1982; Friedman, 1986; Levin, 1992; Pouthas, 1993). How long is an hour? How much of a certain activity can fit within an hour or 20 min? What do I have to do now in order to be ready to leave for school in 10 min? These are the sort of temporal tasks children struggle with and for which they will need support from parents and teachers for many years.

Consequently, when investigating children’s developing sense of time or time-keeping ability, any method involving clock-time is unsuitable. Neither would it be meaningful to look for developmental precursors of clock-time mastery. Since clock-time is such a late contrivance of the human community, we cannot expect to find an innately based capacity for clock-time. Now, someone might object, even though the mechanical clock is of a recent date, time itself has always been the same and the mechanical clock is only a more efficient way of keeping track of it. From our viewpoint of the 21st century this is how it may seem, immersed as we are in standardized, uniform, and abstract clock-time. However, it is a misunderstanding. The introduction of the mechanical clock, meant more than just a more efficient technology, it also introduced a new and different sort of time – a uniform, evenly flowing time. While other modern instruments enabled the detection of previously undetectable natural phenomena such as radiation, the mechanical clock created its own new phenomenon.

## A Short History of Time-Keeping Devices

Temporal organization involving days, months, and years has been around in the human community for millennia. Cyclically recurring celestial events such as the day-night cycle, the cycle of the sun, the cycles of the moon’s phases have informed the construction of calendars since the early days of human communities. It is in principle not too mysterious. The cyclic events are there for the counting, the only requirement is to keep your eyes open and devise a method for keeping track of the cycles. These cyclic natural events then become a back-drop, against which other events may be gaged. This is a simplified description to make a point. In reality, there are records of systematic observations of temporal patterns in the movement of heavenly bodies of all kinds. Not only the most salient, like the sun and the moon. But in principle, even an *uneducated* stone-age man could construct a simple calendar based on these most obvious celestial events.

However, for temporal organization within the day (the 24 h cycle of the earth’s rotation around its axis), there are no natural cycles to count. In ancient civilizations such as Egypt and Babylon sundials and water-clocks were used to aid time-keeping. The principle of the sundial is to subdivide the cycle of the sun into *equal* units. The Egyptians divided the day in 12 h, but these *hours* were not standardized to be uniform the way our modern hours are. They would vary in length with the season. (These unequal hours are sometimes referred to as *temporal hours* or *true time*; Landes, 2000) Thus the temporal units of the sundials could not be used as an objective measure of time, e.g., the duration of an event. They could unambiguously only indicate points in time such as sunrise, high noon, and sunset, which also could be determined simply by eyeballing the sky.

In overcast weather and at night, when the sundial does not work, the water-clock was useful. The principle of the water-clock is different than that of the sundial. Instead of subdividing the duration of a known event (the suns movement across the sky) an event is created (the slow drip of water in or out of a vessel) and then the accumulated *events* (the volume of water) are measured. Interestingly, in antiquity the water-clock was calibrated to conform to the sundial. It had a different scale for different months, even though the technology would easily have allowed for the introduction of standardized time units. This means that, a question such as: *– What time does the sun set?* – would, in ancient Greece, be met with incredulity, and your interlocutor would, while speaking very slowly, explain to you that at sunset the time is SUNSET!

Thus, for most of human history, time-keeping has been a matter of gauging one event against another. Alexander Zsalai’s comment about time in antiquity comes to mind: *“In his time (Heroditus’), and even much later, human activity served much more as a measure of time and not the other way around.”* (Szalai, 1966, described in Levine, 2006). In other words, rather than having time define events, events defined time. This sort of event-time is still in use in some places. If you were to ask a person in rural Burundi when he wants to meet, he might say that he will meet you *when the young cows go out*. In some parts of Madagascar, a question about how long time something takes might produce an answer like *the time of a rice-cooking* (about half an hour; Levine, 2006).

## The Mechanical Clock – A Paradigm Shift in Our Conceptualization of Time

The mechanical clock dates back to the end of the 13th century. The principle of its operation was similar to the water-clock – a uniform, artificial event was generated, and then the event was repeated, while keeping an accumulative count. Unlike the clepsydra, the mechanical clock technology did not allow for calibration with a sundial. It could not handle the ever-changing *temporal hour*. A standard had to be chosen and the choice fell on (subdivisions of) a mean solar-day. The mechanical clocks were at first not very good and they did not indicate minutes. Eventually they improved and in the 17th century when a pendulum was added to the construction, the resulting clock looked like our modern clocks and performed almost as well (Lundmark, 1989; Dorn-van Rossum, 1996; Landes, 2000; NIST, 2009).

The mechanical clock brought about a new sort of time; uniform, objective, and abstract, free of its content. It created uniform units for abstract time. People have always known of an abstract time, beyond or behind the events, i.e., chores could be finished sooner or later, the length of the day varied with the seasons. But without units, abstract time is truly evasive and of little practical use in time-keeping.

## Summary So Far

I think it is safe to say that for time-keeping, the event-time mode has been the standard for a vastly longer period of human existence than has time-keeping by means of clock-time. Therefore I find it unlikely that humans would be equipped with a built-in ability to detect abstract, uniform, and objective time as this sort of time is a product of the mechanical clock. I think it is more likely that our ability to operate with clock-time overlays the older event-time mode. Perceiving abstract, uniform, and objective clock-time is likely a learned skill which entails translating our experience of events into clock-time units.

## Events

One of the most repeated passages, a *Locus Classicus,* in the literature on psychological time, is an anecdote of how events sometimes distort our estimation of time. It typically reads something like this: – *Have you noticed how, when you are engaged in or observing a rousing, entertaining or novel event, time seems to pass rapidly, while time seems to drag when nothing much is happening or the event is a boring one.* Generally the analysis ends there. The assumption seems to be that the temporal information embedded in events is inherently unreliable, and events are therefore rejected as a source of temporal information. I think this rejection may be a bit premature. Undoubtedly, there are extraordinarily captivating events which make us forget about everything else, as well as sluggish ones that never seem to end, but these are at the extreme ends of the scale. There are also events somewhere in the middle, appealing or important enough to keep your attention up, but not so to make us lose sight of other matters of the day.

Of particular interest for the account presented here is a class of events which we have experienced many times, and regarding which we possess a substantial amount of knowledge or event-knowledge. These are the events and activities of everyday life which are so familiar to us that the memories of them come to possess a schema- or script-like character. This type of events are frequently referred to as *everyday events, routine events,* or *recurring events*. Given that it is logically impossible for the same event to happen more than once, our minds are apparently not conforming to the rules of logic in this matter. This is more than a lucky accident, since our event-scripts are so useful to us. An event-script may scaffold our memory so that we don’t have to remember everything from scratch; we know how the type of event usually unfolds. It guides our perception and attention so that we may interpret a situation quicker; we know what to look for. If we know how an event usually unfolds, we may make better predictions about what will happen next and what actions to take. (Zacks et al., 2007; Sargent et al., 2013). Furthermore, it is a matter of cognitive ergonomy; to process a routine event requires less resources than if we had to perceive or interpret it from scratch, as a novel event, every time. This way we may reserve resources for dealing with unexpected and perhaps dangerous occurrences.

As the event scripts are acquired through individual experience, we might expect a certain amount of variation between individuals. And there are differences, but also a surprisingly good agreement between individuals regarding what constituent parts makes up a certain type of event (Bower et al., 1979), and between and within individuals in how events are temporally structured (Newtson, 1976; Zacks and Tversky, 2001; Speer et al., 2003). The consistency in how we perceive everyday events implies that our experience can be communicated and reasoned about together with others, which is very helpful in temporal organization endeavors.

## Children and Events

Contrary to the traditional belief that young children’s skills are poor in representing and remembering an event sequence (Piaget, 1926, 1969; Fraisse, 1963), Nelson and Gruendel (1981), found that even quite young children have generalized, temporally organized representations of familiar, everyday events (Nelson, 1986, 1996). Children, as young as 3 years, can when asked about familiar events, such as going to a birthday party or having lunch at their preschool, verbally report the component acts of these events in correct temporal order. And already at the age of 4, children begin to grasp temporal relations among everyday events, such as waking, eating lunch, eating dinner, and going to bed (Friedman, 1977, 1982, 1990). Young children accomplish these tasks with the help of script-like event representations. The event scripts help them predict the course of events in everyday life as well as guiding action and attention; they serve as representation of past experience, and helps with the interpretation of present experience of events.

For children the event scripts also have a more profound function as they may be the child’s earliest form of knowledge representation and as such a basic building block of cognition that serve as a foundation for more complex cognitive structures (Nelson and Gruendel, 1981, p. 150, 155; Lucariello and Rifkin, 1986). Children’s event representations eventually give rise to more abstract forms of knowledge, such as concepts and categories and also support language acquisition. Logical and temporal relations first appears in the context of event representations. Time and temporal relations “is a basic dimension of action, activity, and event structure” (Nelson, 1996, p 259) and thus part of the child’s experience of events. Two basic dimensions of time, duration, and sequence (e.g., Fraisse, 1963), are also basic and indispensable dimensions of events. In turn, these basic dimensions embed other time concepts such as *before and after*, *while, now*, and *soon.* Thus, a basic understanding of temporal relations is implicit in the young children’s knowledge of events. The trick that children are expected to, and eventually come to master is to translate their event experience into clock-time and its linguistic representations (Nelson, 1996). In Nelson’s view, language is crucial to children’s development of time knowledge because language is an important mediator of knowledge. Language makes it possible to construct abstract concepts and complex representations that go beyond the more basic ones acquired from direct experience of events (Nelson, 1996).

I agree with Nelson, but I would like to add that perhaps the experience and understanding of events also have a more direct effect on children’s emerging knowledge of time, as our primordial mode of experiencing and understanding time may be by way of events.

## What Events?

In psychology and philosophy events are sometimes defined as simply a change (e.g., Rey, 2015)(Ducasse, 1926; von Wright, 1963, described in Casati and Varzi, 2014). Though, for the time-keeping discourse presented here, a single change doesn’t qualify as an event. Neither does a series of unrelated changes, although they may (or may not) give rise to some sort of temporal experience. To be functional in time-keeping, the event must consist of a series of related changes, i.e., a coherent everyday event with a beginning, a middle, and an end. This sort of event is perceived as a unit because it has a meaning; a purpose or an end state. A broad and informal definition would be *Go-together goings-on.* Most importantly, this is the sort of events which make up much of everyday life and from which we may form event-scripts and event configuration scripts.

A change could be part of this event but any random change does not necessarily constitute a time-keeping event. Thus, this sort of event contains not only changes, but also continuity. In everyday life we experience events such as going to work, cleaning up after dinner, playing a game of soccer. Events of this type and on this scale are the ones we need to choreograph as we maneuver through an ordinary day in real life. Consequently it is events of this sort and on this scale that are of interest here. Katherine Nelson’s description captures the gist of everyday events well: “…they involve people in purposeful activities, and acting on objects and interacting with each other to achieve some result” (Nelson, 1986, p 11). Thus, the meaning of *event* in this article has more in common with its meaning in everyday language than with its meaning in Philosophy or any other academic discipline.

The aim of the research described above was not to uncover the processes underlying time-keeping, but I think these results indicate that event representations play an important role in everyday life and furthermore, that events are not merely random noise but are perceived in a consistent and lawful manner. This in turn suggests that event-time and time-keeping by way of events possibly still is part of our cognitive repertoire. In my view, our modern way of time-keeping most likely consists of event-time together with clock-time. Children may, however, rely more on event-time and it may therefore be advantageous to investigate the development of time-keeping ability or sense of time in the context of events.

## Event Based Time-Keeping

### So What Would a Time-Keeping Task by Means of Event-Time be Like? The Figure (Figure 1) Shows an Example

Reasoning in terms of temporal relations among events entails a sort of mental time-travel. By constructing a mental event-model we may stop time for a moment, so that we may, in our minds, travel forward in time, and also backward to try out different alternatives. As some of the events overlap, we must also travel sideways.

**FIGURE 1:**
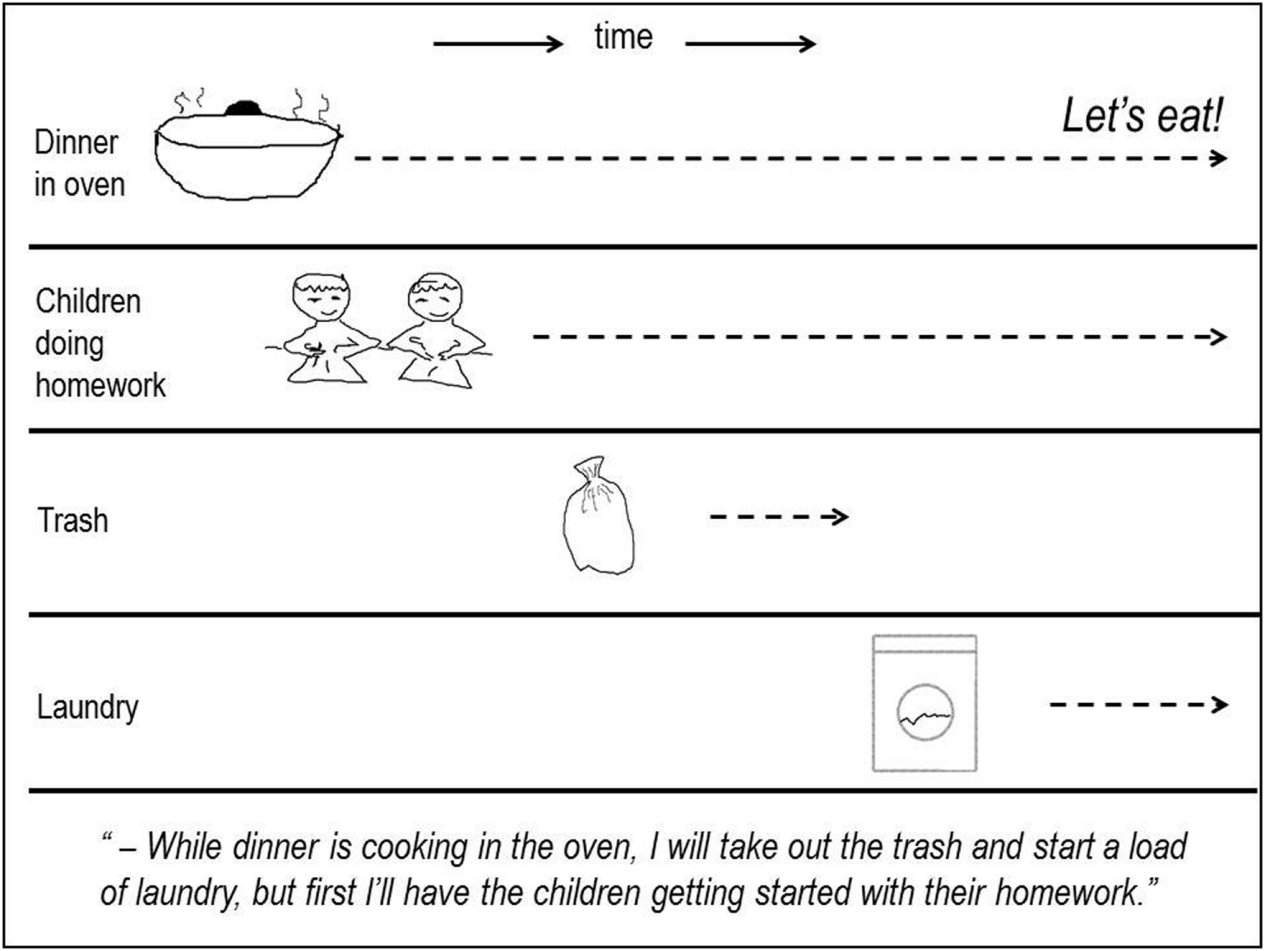
**Mental event-model of temporal relations among four events**.

With their greater repertoire of event-representations and greater general processing resources, adults are obviously more competent event based time-keepers than children. In my opinion, it is the precursors of this competency we should look for when investigating children’s sense of time.
